# Antifungal potential of isoespintanol extracted from *Oxandra xylopioides* diels (Annonaceae) against intrahospital isolations of *Candida* SPP

**DOI:** 10.1016/j.heliyon.2022.e11110

**Published:** 2022-10-18

**Authors:** Orfa Inés Contreras Martínez, Alberto Angulo Ortíz, Gilmar Santafé Patiño

**Affiliations:** Universidad de Córdoba, Facultad de Ciencias Básicas, Montería, Córdoba, Colombia

**Keywords:** Isoespintanol, *Oxandra xylopioides*, *Candida* spp., Antifungal

## Abstract

The aim of this study was to evaluate the antifungal activity of isoespintanol (ISO) extracted from *Oxandra xylopioides* Diels (Annonaceae) against clinical isolates of *Candida* spp. Isoespintanol was obtained from the petroleum benzine extract of the leaves and was identified by nuclear magnetic resonance (NMR) and mass spectrometry (MS). For antifungal activity experiments, the broth microdilution method was used. The results show an inhibitory effect against *Candida* spp., with minimum inhibitory concentration (MIC) values between 450.4–503.3 μg/mL. Furthermore, the inhibitory effect of ISO against fungal biofilms is highlighted, even in some cases, greater than the effect shown by amphotericin B (AFB) and in others, where AFB showed no effect. Assays with fluorescent staining with acridine orange (AO) and ethidium bromide (EB), transmission electron microscopy (TEM), Evans blue, measurement of extracellular pH and leakage of intracellular material, evidenced damage at the level of fungal membranes and general cell damage, when cells were exposed to ISO, compared to untreated cells. The results of this research, serve as the basis for future studies in the establishment of the mechanisms of antifungal action of ISO, which could serve as an adjunct in the treatment of infections by these yeasts.

## 1 Introduction

Fungi are a major cause of opportunistic infections. They infect countless numbers of people each year, raising morbidity and mortality rates, especially in immunocompromised people; in recent years, these infections have garnered significant attention in medical and pharmaceutical science, due to the rapid increase in its incidence. Currently, more than one billion people are affected by fungal infections (Kakar et al., 2021) and more than 1.5 million deaths are produced per year (Bongomin et al., 2017). Recent studies estimate that infections caused by fungi, which especially include *Candida* spp., are difficult to treat and the associated mortality remains very high, even when antifungal treatments are available (Janbon et al., 2019). More than 90% of people infected with HIV develop debilitating diseases caused by species of the genus *Candida*, these being the most common pathogens isolated from patients in intensive care. Candidemia is one of the most frequent opportunistic mycoses in the world, *C. albicans* is considered the most prevalent and clinically important pathogenic yeast (Kakar et al., 2021). However, an important epidemiological change has been observed in recent decades, species such as *C. glabrata*, *C. tropicalis*, *C. krusei*. *C. parapsilosis*, *C. auris*, have begun to be isolated with frequencies in severe candidemia around the world (Donadu et al., 2021). The increasing number of patients at risk of invasive mycosis has a complex management, since drug interactions, intolerance of currently available antifungals and innate or acquired resistance pose a problem for their treatment, which is restricted to five established classes of antifungal medications; The Centers for Disease Control and Prevention (CDC) further predicts that these infections pose a threat that will worsen and may become urgent (Scorneaux et al., 2017). In this context, the search and development of new compounds with antifungal potential, which are more effective and safe, as well as the development of new treatment strategies with better host tolerance has become a priority objective today (Gintjee et al., 2020).

Natural products play a valuable role in the discovery and development of many drugs used today; especially plants play a main role as a source of specialized metabolites with recognized medicinal properties (Aylate et al., 2017). Due to their wide chemical diversity, these metabolites can be used directly as bioactive compounds, as drug prototypes and/or be used as pharmacological tools for different targets (Avato, 2020). About 80% of the world's population use herbal treatments for health care (Mekonnen Bayisa and Aga Bullo, 2021); These have been used in traditional medicine and in the pharmaceutical industry for a long time due to their invaluable therapeutic potential (Naman et al., 2016). ISO (2,5-dimethoxy-3-hydroxy-*p*-cymene), is a monoterpene known for its anti-inflammatory activity ((Rojano et al., 2007, Rojano et al., 2007), vasodilator (Rinaldi et al., 2019), antispasmodic (Gavilánez et al., 2018), with a high antioxidant potential (Rojano et al., 2008). Its insecticidal activity against *Spodoptera frugiperda* (Rojano et al., 2007, Rojano et al., 2007) and antifungal against *Colletrotichum* (Arango et al., 2007) have also been tested. However, its potential against clinical isolates has not been reported. The purpose of this study was to investigate the antifungal effect of ISO against clinical isolates of *Candida* species and its effect on the inhibition of fungal biofilms. The results highlight the antifungal activity of ISO against clinical isolates of *Candida* spp., probably due, at least in part, to damage to the integrity of fungal cell membranes, resulting in the alteration of its permeability and the consequent loss of intracellular material; furthermore, we highlight the role of ISO in the inhibition of fungal biofilms.

## 2 Materials and methods

### 2.1 Reagents

Fluconazole (FLC) was obtained from Pfizer. Amphotericin B, acridine orange, ethidium bromide, Evans blue, sabouraud dextrose broth (SDB) and sabouraud dextrose agar (SDA) used in this study were obtained from Sigma-Aldrich. Glacial acetic acid from Carlo Erba Reagents, Italy.

### 2.2 Vegetal material

The ISO was isolated from leaves of *O. xylopioides*, which were collected from a specimen located in the Municipality of Montería, Department of Córdoba, with coordinates 08° 48′17″ north latitude and 75°42′07″ west longitude. A specimen of herbarium is deposited in the Joaquín Antonio Uribe Botanical Garden, with the collection number JAUM 037849.

### 2.3 Isolation and identification of isoespintanol

The ISO was obtained by hydrodistillation and crystallization, from 15 g of petroleum benzyne extract of the leaves of *O. xylopioides*, following the methodology described in a previous work (Ramírez et al., 2015), with some modifications that included successive crystallizations with n-hexane that led to obtaining 3.92 g of the pure compound. The chromatographic monitoring was done with aluminum thin layer chromatography TLC plate, silica gel coated with fluorescent indicator F254 (Merck®). The purity was verified using a gas chromatograph coupled to a Thermo Scientific model Trace 1310 mass spectrometer, with an AB-5MS column, (30 m × 0.25 mm i.d. × 0.25 μm). The temperature gradient system started at 80 °C for 10 min (min) up to 200 °C at 10 °C/min. The temperature was increased to 240 °C at 4 °C/min and finally it was brought up to 290 °C for 10 min at 10 °C/min. The injection was splitless type, with an injection volume of 1 μL. The mass spectrum was obtained in electron impact ionization mode at 70 eV. The structure of the ISO was determined using ^1^H-NMR, ^13^C-NMR, DEPT, COSY ^1^H–^1^H, HMQC and HMBC spectra, performed on a 400MHz Bruker Advance DRX spectrometer, in deuterated chloroform (CDCl_3_). The ISO was purified as a creamy white amorphous solid. The EI-MS: [M] ^+^ m/z 210 (49%) and fragments m/z 195 (100%), 180, 165, 150, 135 and 91. ^1^H-NMR (CDCl_3_): δ 6.22 s, 1H (H6), δ 5.85 s, 1H (HO-3), δ 3.77 s, 3H (H12), δ 3.76 s, 3H (H11), δ 3.52 hep, J = 7.1 Hz, 1H (H8), δ 2.29 s, 3H (H7), δ 1.33 d, J = 7.1 Hz, 6H (H9–H10). ^13^C-NMR (CDCl_3_): δ 154.3 (C5), δ 147.4 (C3), δ 139.7 (C2), δ 126.8 (C1), δ 120.4 (C4), δ 104.4 (C6), δ 24.6 (C8), δ 60.8 (C11), δ 55.7 (C12), δ 20.6 (C9, C10), δ 15.8 (C7).

### 2.4 Tested microorganisms

Fifteen clinical isolates belonging to *Candida* spp., including: *C. albicans*, *C. glabrata C. tropicalis*, *C. auris*, were used in this study. The isolates were cultured from tracheal aspirate samples, blood cultures and urine cultures from hospitalized patients at the Salud Social S.A.S. from the city of Sincelejo, Sucre, Colombia. All microorganisms were identified by standard methods: Vitek 2 Compact. Biomerieux SA., YST vitek 2 Card and AST-YS08 Vitek 2 Card (Ref 420739). The medium SDA, was used to maintain the cultures until the tests were carried out.

### 2.5 Antifungal susceptibility assay

Minimum inhibitory concentration (MIC) of the ISO against the clinical isolates of *Candida* spp., was defined as the lowest concentration at which 90% (IC_90_) of the fungal growth was inhibited, compared to the control. IC_50_ was defined as the lowest concentration at which 50% of fungal growth was inhibited. The MIC was determined by performing the microdilution assay in broth, using 96-well microtitration plates (Nunclon Delta, Thermo Fisher Scientific, Waltham, MA, USA), as described in the method M27-A3 of the Clinical Laboratory Standards Institute (CLSI) (Cantón et al., 2007) and EUCAST (European Committee for Antimicrobial Susceptibility Testing) (Rodriguez-Tudela, 2003), with minor modifications. Serial dilutions were made in SDB to obtain final concentrations of 15.62–500 μg/mL of the ISO in each reaction well. The assays were developed at a final volume of 200 μL per well as follows: 100 μL of the fungal inoculum at a concentration of 1 × 10^5^ CFU/mL read at 530nm on a Spectroquant® Prove 300 spectrophotometer and 100 μL of the ISO adjusted to achieve in a final reaction system the concentrations described above. Yeast isolates without ISO and with FLC were used as controls. The plates were incubated at 37 °C for 24 h. The experiments were carried out in triplicate. The inhibition of fungal growth by ISO was determined by change in optical density (OD) using a SYNERGY LX microplate reader (Biotek), at 590 nm, from the beginning of the incubation to the final moment (24 h) and the reduction percentage growth was calculated (Quave et al., 2008). The OD_590_ value of the untreated cells was assigned 100% growth. Subsequently, the minimum fungicidal concentration (MFC) was determined by taking 10 μL from each well and inoculating it on SDA. The plates were sealed and incubated at 37 °C for 24/48 h, checking for microbial growth. MFC was considered the lowest concentration capable of inhibiting 99% of yeasts (Donadu et al., 2021). The experiments were carried out in triplicate.

### 2.6 Live/Dead assay

The LIVE/DEAD assay were developed following the methodology proposed by (Zhang et al., 2020) with some modifications. The yeast suspension was previously standardized (1–5 × 10^6^ CFU/mL), then sterile coverslips were placed in six-well plates and 2 mL of the fungal solution was added to each well. After incubation for 24 h, the cells were washed three times with phosphate-buffered saline (PBS). The ISO and FLC at MIC concentration for each yeast were added to the experimental groups and the fungal inoculum in SDB was used as a control. The prepared six-well dishes were incubated at 35 °C for 24 h. Then the coverslips were washed three times with PBS. Acridine orange (AO) (5 μL, 100 mg/L) and ethidium bromide (EB) (5 μL, 100 mg/L) were mixed under dark conditions, then the AO/EB mixture was added to the coverslips in dark conditions for 30 s. An Olympus BX43 fluorescence microscope with DP72 camera was used to observe and photograph.

### 2.7 Transmission electron microscopy (TEM)

The morphology of *C. albicans* after ISO treatment was analyzed through TEM. The *C. albicans* concentration was adjusted to 1 × 10^6^ CFU/mL; the suspension was mixed with ISO (200 μg/mL) and incubated at 37 °C for 24 h. Subsequently, the cells were collected and fixed in 2.5% glutaraldehyde at 4 °C; they were centrifuged at 13000 rpm for 3 min and the button at the bottom of the vial was post-fixed in 1% osmium tetroxide in water, for 2 h at 4 °C. Then pre-imbibition with 3% uranyl acetate was performed for 1 h at room temperature, then the cells were dehydrated in an ethanol gradient (50% for 10 min, 70% for 10 min, 90% for 10 min, 100% for 10 min), acetone-ethanol (1:1) for 15 min and embedded in SPURR epoxy resin. The samples were cut on a Leica EM UC7 ultramicrotome, at 130 nm thick and contrasted with 6% uranyl acetate and lead citrate, finally they were observed in a JEOL 1400 plus transmission electron microscope. The photographs were obtained with a Gatan Orius CCD camera.

### 2.8 Quantitative evaluation of biofilm formation

The standardized samples of *C. albicans*, *C. glabrata*, *C. tropicalis*, *C. auris* were evaluated to quantify the reduction of biofilms in the presence of ISO in plates of 96 wells following the methodology reported by (Donadu et al., 2021) with some modifications. For biofilm formation, 200 μL of the samples were cultured in each well in SDB and incubated at 37 °C for 48 h. Then the broth was removed from the microplate and 200 μL of the ISO MIC for each isolate were added and incubated at 37 °C for 1 h. Then the floating bacteria were removed and the biofilm at the bottom of the wells was washed with deionized water three times. Six replicas of each sample were made. The cultures without ISO were taken as a control and AFB was used as a positive control, a drug with known efficacy against biofilms in *Candida* spp. (Mukherjee et al., 2009). Biofilm reduction was quantified by staining the wells with 0.1% crystal violet (Sigma-Aldrich) for 20 min. The samples were washed with deionized water until the excess of dye was removed. Finally, the samples were soaked in 250 μL of 30% glacial acetic acid (Carlo Erba Reagents).

Absorbance values at 590 nm (OD_590_) were measured for each strain using a SYNERGY LX microplate reader (Biotek). Biofilm production was grouped into the following categories: OD_590_ < 0.1: non-producers (NP), OD_590_ 0.1–1.0: weak producers (WP), OD_590_ 1.1–3.0: moderate producers (MP) and OD_590_ > 3.0: strong producers (SP). Biofilm reduction was calculated using the following equation:

$${\%\;\text{reduction}\;\text{of}\;\text{biofilms}:\;\text{Abs}}_{\text{CO}}-{\text{Abs}}_{\text{ISO}}{/\text{Abs}}_{\text{CO}}\times 100$$

where, Abs_CO_: absorbance of the control sample and Abs_ISO_: absorbance of the sample treated with ISO.

### 2.9 Release of cellular material through the fungal membrane

The release of cellular constituents in the supernatants was measured according to the methodology proposed by (Tao et al., 2014) with some modifications; 20 mL of fungal culture in SDB was centrifuged at 4000 g for 20 min, washed 3 times and resuspended in 20 mL of PBS (pH 7.0). The suspension was then treated with ISO (MIC) and incubated at 37 °C for 0, 30, 60 and 120 min of treatment. Subsequently, 2 mL of the sample were collected and centrifuged at 4000 g for 20 min. To determine the concentration of the released constituents, 2 ml of supernatant was used to measure the absorbance at 260 nm with the Spectroquant® Prove 300 UV/Vis spectrophotometer. Samples without ISO and samples with FLC were used as controls.

### 2.10 Extracellular pH measurement

The extracellular pH of the *Candida* spp. treated with the ISO (MIC) was determined according to the methodology proposed by (Tao et al., 2014) with some modifications. 100 μL of the fungal suspension (1 × 10^5^ CFU/mL) was added to 20 mL of SDB and incubated at 37 °C for 48 h. Subsequently, the samples were centrifuged at 4000 g for 20 min; the pellet was collected, resuspended, and washed three times with double distilled water and resuspended again in 20 mL of sterile double distilled water. After the addition of ISO (MIC), the extracellular pH of *Candida* spp., was determined at 0, 30, 60 and 120 min, using a Schott® Instruments Handylab pH 11 m. Untreated samples and samples with FLC were analyzed as control.

### 2.11 Effect of ISO on membrane integrity

Damage to yeast cell membranes produced by ISO was also evidenced using Evans blue staining (Sigma-Aldrich) according to the methodology proposed by (Chaves-Lopez et al., 2018) with some modifications. Before assay, Evans blue was prepared 1% in PBS. 100 μL of the fungal suspension in SDB were incubated on coverslips (22 mm × 22 mm) in triplicate at 37 °C for 24 h. Subsequently, the samples were treated with ISO (MIC) for 1 h, and then, 1 mL of Evans blue was added to the samples for 5 min. Cells not treated with ISO were used as a control. The samples were observed under the microscope, Olympus CX31.

### 2.12 Statistical analysis

The results were analyzed using the statistical software R version 4.1.1. Pearson's correlation coefficient was used to measure the degree of linear relationship between the ISO concentration and the percentage reduction in microbial growth. To compare the effects of ISO and AFB on the reduction of the percentage of biofilms, the Mann-Whitney U Test was used. The Student's T test was applied to compare the effects of ISO and FLC on the exit of intracellular material through the membrane (reading OD_260_ nm). The one-factor analysis of variance was used to compare the effects of the treatments on the extracellular pH and the honest Tukey test was used to evaluate the significant differences produced by the analysis of variance.

## 3 Result and discussion

### 3.1 Obtaining isoespintanol

**Figure 1:**
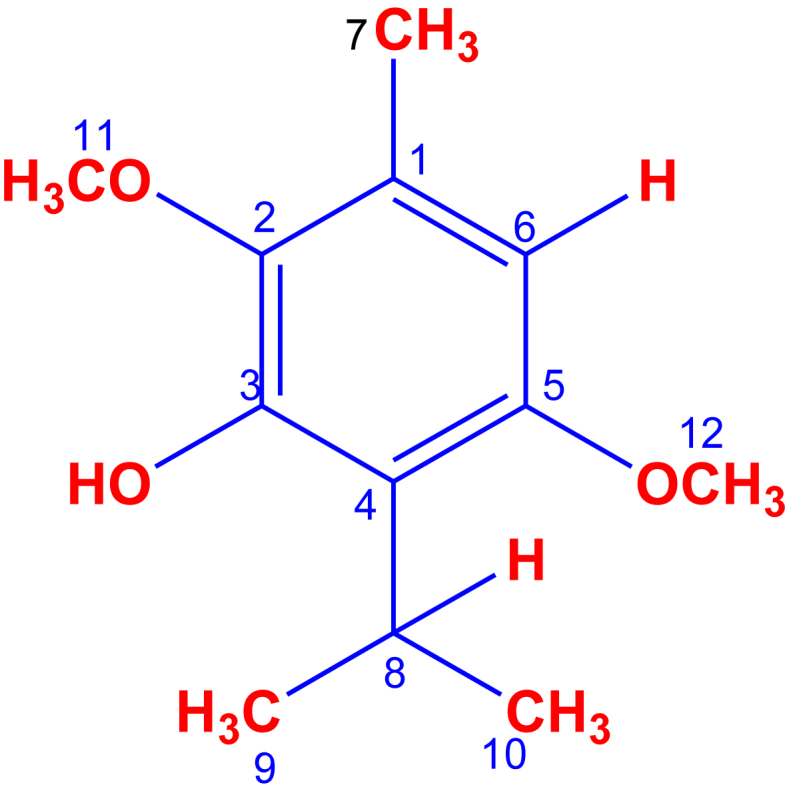
Structure of isoespintanol

ISO (3.92 g) were obtained from 15 g of petroleum benzyne extract of the leaves of *O. xylopioides*, with a purity greater than 99%, verified by GC-MS. Its structural identification by ^1^H-NMR, ^13^C-NMR, DEPT, COSY ^1^H–^1^H, HMQC and HMBC, led unequivocally to propose the structure of 2,5-dimethoxy-3-hydroxy-*p*-cymene, isoespintanol, Figure 1.

### 3.2 Antifungal susceptibility assay

**Figure 2:**
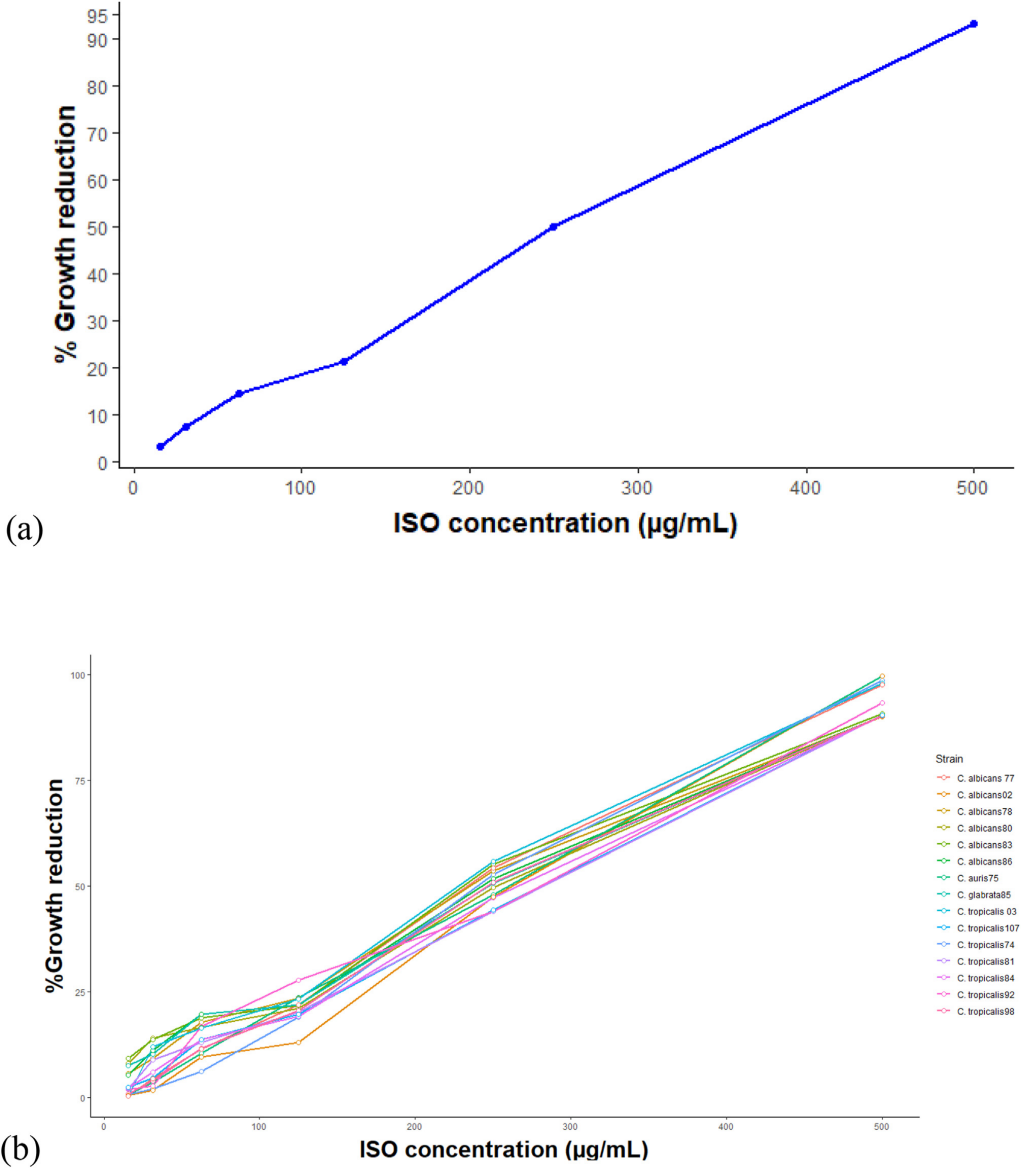
Percentages of reduction of the fungal growth of *Candida* spp., in the presence of different concentrations of the ISO. In (a) positive linear relationship between ISO concentration and growth reduction percentage. In (b), a similar trend between each of the isolates as the percentage of growth reduction increases as the ISO concentration increases.

Our results show that ISO extracted from *O. xylopioides* has antifungal activity against clinical isolates of *Candida* spp. When *C. albicans*, *C. tropicalis*, *C. glabrata* and *C. auris* are exposed to ISO, a reduction in cell growth is observed compared to the untreated strains used as controls. In Figure 2(a), the strong and positive linear relationship between the ISO concentration and the growth reduction percentage is evidenced, as can be seen, as the ISO concentration increases, the percentage of microbial growth reduction also increases, which coincides with Pearson's correlation coefficient (0.998). Furthermore, the hypothesis test on the correlation coefficient yields a p-value < 0.05, which indicates that with 95% confidence, there is a significant linear relationship. In Figure 2(b), the similar tendency between each of the isolates is shown to increase the percentage of growth reduction as the ISO concentration increases. It should be noted that all clinical isolates of *Candida* spp., in this study were resistant to FLC. Table 1 shows the ISO IC_90_, IC_50_ and MFC values against *Candida* spp. Our research highlights the antifungal effect of ISO against *C. auris* (IC_50_: 257.4–IC_90_: 453.5 μg/mL) an emerging pathogen with a high percentage of mortality associated with therapeutic failure (Carvajal et al., 2021); *C. glabrata* (IC_50_: 261.7–IC_90_: 496.0 μg/mL) considered the second most isolated *Candida* species, associated with invasive candidiasis after *C. albicans* and resistant to antifungal drugs, particularly FLC and other azole derivatives (Hassan and Chew, 2021) and *C. tropicalis* (with IC_90_ values between: 450,4 and 500.7 μg/mL) one of the most important non-albicans species with resistance to azoles (Chen et al., 2021), showing MIC values with an increasing trend towards high MICs, which is consistent with studies reported by (El-Kholy et al., 2021). The MIC and MFC of the ISO was between 450.4–503.3 μg/mL (Table 1), these values varied between species and within the same *Candida* species. The MFC values for all isolates were the same for MIC; being the concentration of the ISO determining in the degree of sensitivity of the species of *Candida*. Our results are consistent with studies reported with other monoterpenes with a chemical structure similar to ISO, such as thymol (Dias de Castro et al., 2015), and carvacrol (Oliveira Lima et al., 2013), to which, antifungal activity against *Candida* spp., has been reported, apparently by binding to ergosterol in the fungal membrane, altering the permeability of the membrane and causing cell death. Citral has also been reported with antifungal activity against *Penicillium italicum*, due to damage to the integrity of the membrane and increased permeability as reported by (Tao et al., 2014). Studies with eugenol (De Oliveira et al., 2013) and linalool (De Oliveira Lima et al., 2017) have also shown antifungal activity, showing action on the *Trichophyton rubrum* membrane through a mechanism that seems to involve the inhibition of biosynthesis ergosterol, with MIC values similar to those found in our results; in general, numerous terpenes are known to be active against a wide variety of microorganisms (Murphy Cowan, 1999), however, unlike our research, the activity of ISO against fungal clinical isolates has not been reported.

**Table 1:** Minimum inhibitory concentration (IC_90_ μg/mL), IC_50_ and minimum fungicidal concentration of the ISO against *Candida* spp., After 24 h of treatment.

Fungi	Isoespintanol (μg/mL) 24 h		
	IC_50_	IC_90_	MFC
*C. albicans 02*	266,9 ± 0,01	459,2 ± 0,01	459,2
*C. albicans 77*	254,8 ± 0,02	452,4 ± 0,02	452,4
*C. albicans 78*	259,7 ± 0,01	488,4 ± 0,01	488,4
*C. albicans 80*	263,2 ± 0,30	499,8 ± 0,30	499,8
*C. albicans 83*	252,9 ± 0,02	487,9 ± 0,02	487,9
*C. albicans 86*	261,1 ± 0,03	493 ± 0,03	493
*C. tropicalis 03*	245 ± 0,01	450,4 ± 0,01	450,4
*C. tropicalis 74*	260,7 ± 0,3	452,1 ± 0,3	452,1
*C. tropicalis 81*	278,2 ± 0,02	503,3 ± 0,02	503,3
*C. tropicalis 84*	276,1 ± 0,02	497,0 ± 0,02	497,0
*C. tropicalis 92*	266,6 ± 0,02	483,3 ± 0,02	483,3
*C. tropicalis 98*	273,5 ± 0,02	487,9 ± 0,02	487,9
*C. tropicalis 107*	279,5 ± 0,04	500,7 ± 0,04	500,7
*C. glabrata 85*	261,7 ± 0,03	496,0 ± 0,03	496,0
*C. auris 75*	257,4 ± 0,29	453,5 ± 0,29	453,5

The MIC (IC_90_) was defined as the lowest ISO concentration that reduced fungal growth (≥90%) compared to untreated cells used as controls.

### 3.3 Live/Dead assay

**Figure 3:**
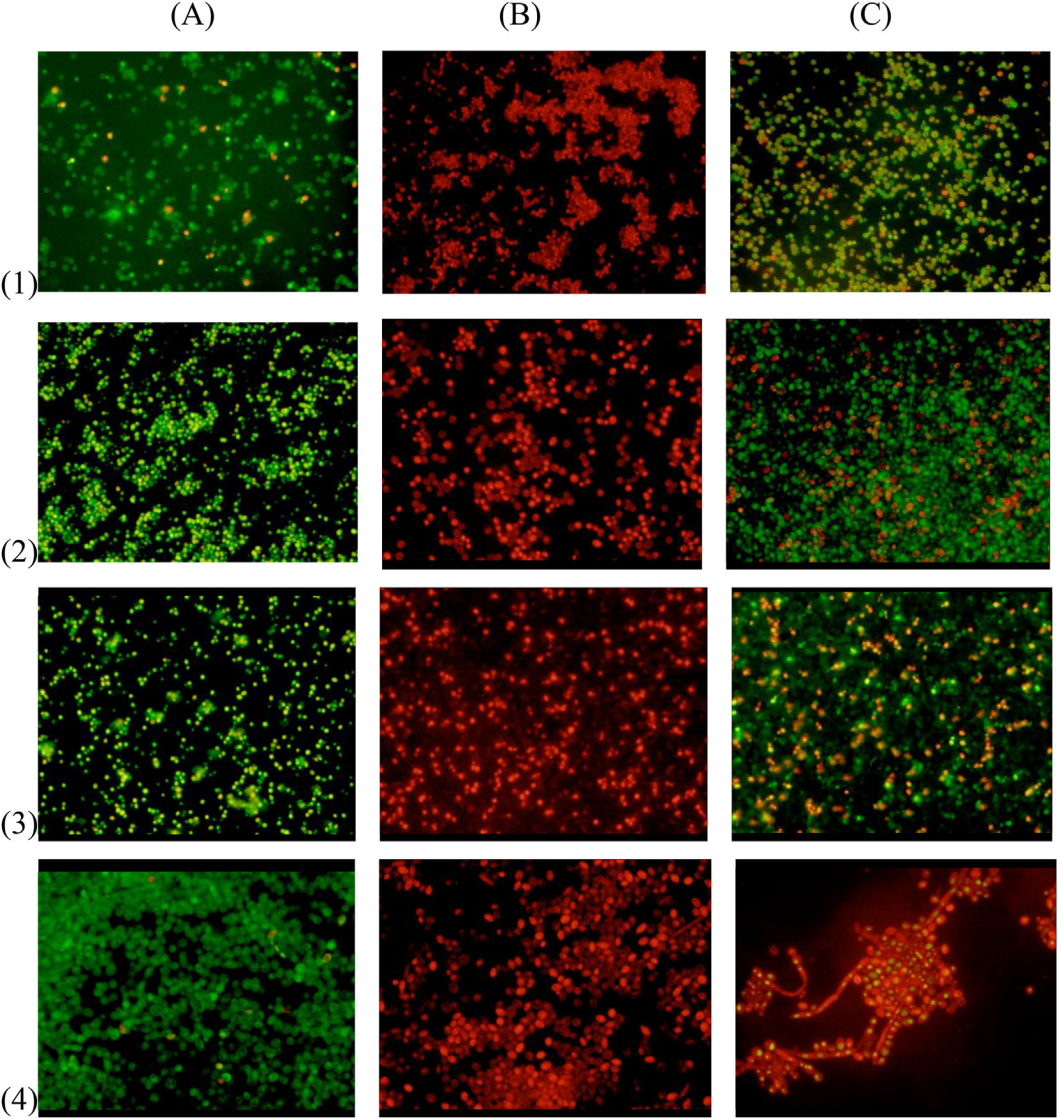
Fluorescence microscopy of *Candida* spp., Treated with ISO (MIC μg/mL) and FLC after 24 h. Cells were stained with OA/EB. *C. auris* (1) *C. albicans* (2), *C. glabrata* (3) and *C. tropicalis* (4). In column (A) untreated cells, (B) ISO treated cells and (C) FLC treated cells. Live cells appear green and dead cells red with double fluorescent staining.

After double fluorescent staining with AO and EB, the morphology of the cells was observed under fluorescence microscopy. In living cells, AO diffuses through intact cytoplasmic membranes (Byvaltsev et al., 2019), intercalates with DNA, and emits a bright green fluorescence (Zhang et al., 2020). However, EB penetrates only dead cells with compromised cell wall and membrane systems (Wu et al., 2015), intercalates with DNA, and emits a red-orange fluorescence. In our results (Figure 3), untreated cells grew well after 24 h (A), while dead cells were observed massively in the group treated with ISO (B) and to a lesser extent in the group treated with FLC (C); These results are probably due to damage to the integrity of the membranes of cells treated with ISO.

### 3.4 Transmission electron microscopy (TEM)

**Figure 4:**
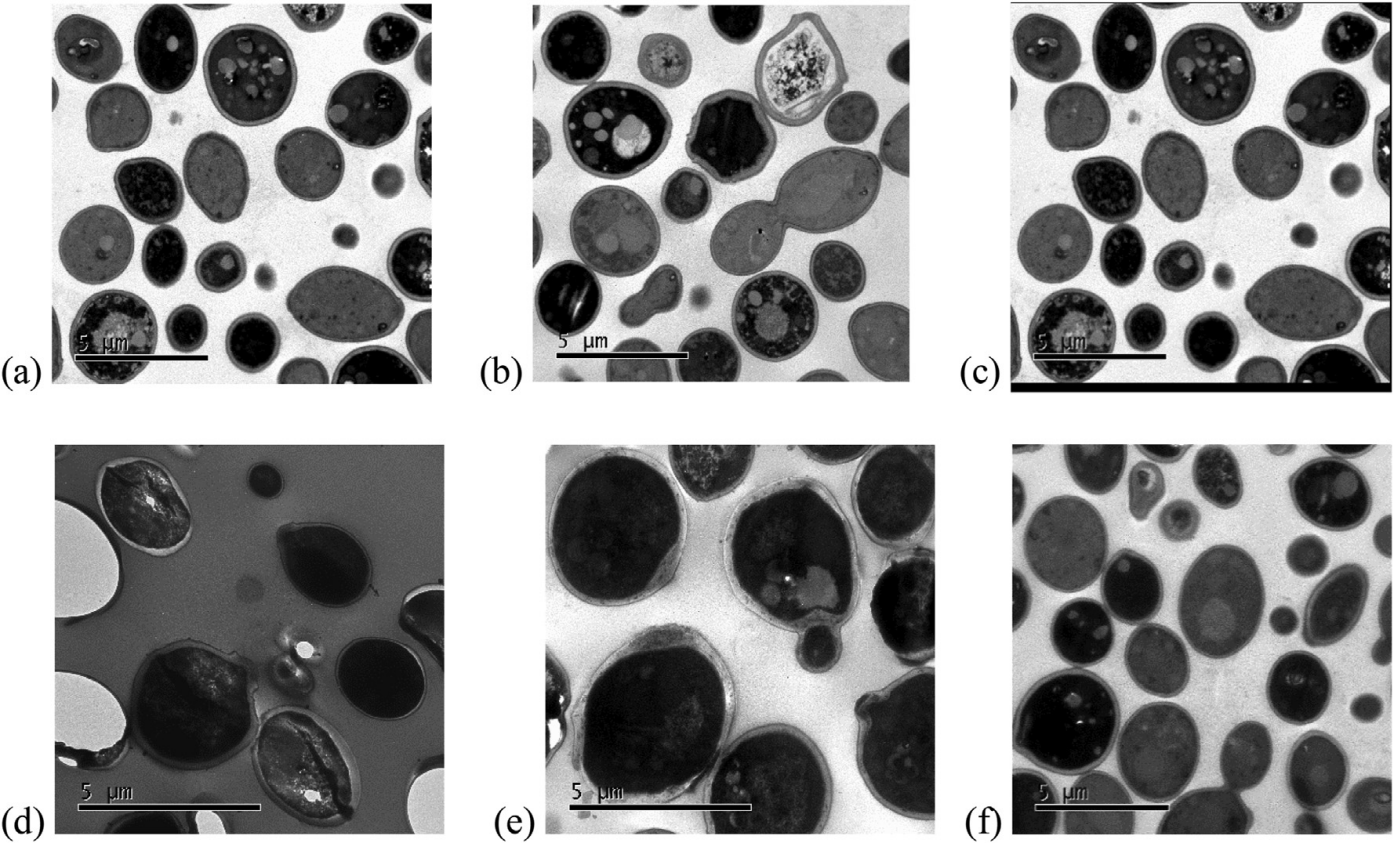
TEM analysis of *C. albicans*. (a, b, c) *C. albicans* treated with ISO. (d) *C. albicans* without treatment. (e and f) *C. albicans* treated with FLC.

The images (Figure 4) show the irregular morphology of some *C. albicans* cells 24 h after ISO treatment. In some cells, the cell membrane looks discontinuous, partially dissolved and irregular regions unlike cells not treated with ISO. However, some cells with apparently intact membranes show a cellular disorganization at the cytoplasm level with intense vacuolization and nuclear fragmentation, this could indicate that ISO can cross the membrane, penetrating inside the cells and interacting with specific intracellular sites. which could also be responsible for its antimicrobial activity, taking into account that studies reported by (Trombetta et al., 2005) suggest a real transfer of monoterpenes through lipid bilayers; the discrepancy with what was observed with cells treated with fluconazole could indicate that there are other molecules or other target sites, in addition to the membrane, that determine the sensitivity of *Candida* species to ISO, so it could be speculated that the mechanism of action of the ISO could also be associated with other fungal cell target sites as indicated (Cheng et al., 2021). The cells treated with FLC, some remained normal with intact cell walls, the damage at the membrane level and at the level of the cytoplasm was less than that observed in cells treated with ISO.

### 3.5 Quantitative evaluation of biofilm formation

**Figure 5:**
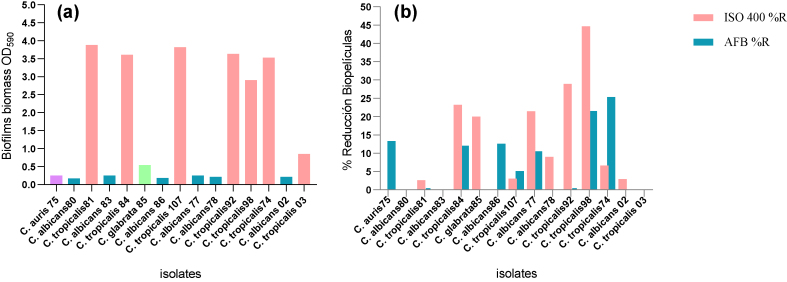
Effect of ISO on *C. auris*, *C. albicans*, *C. glabrata* and *C. tropicalis*. (a) shows the formation of biofilms at 37 °C for 48 h. (b) shows the percentage of biofilm reduction after 1 h of treatment with ISO (MIC μg/mL) and AFB (1.0 μg/mL).

The formation of fungal biofilms increases the persistence and dissemination ability of yeasts, especially in hospital environments (Hassan and Chew, 2021). Biofilm-forming *Candida* strains are associated with high mortality, probably due to the low permeability of the matrix to antifungal drugs (Tascini et al., 2017). In our study, all isolates were biofilm producers (Figure 5(a)), classified as follows: OD_590_ < 0.1: no production, OD_590_ 0.1–1.0: weak production, OD_590_ 1.0–3.0: moderate production and OD_590_ > 3: strong production; highlighting the *C. tropicalis* isolates for presenting higher biofilm biomass, compared to the other species. This is consistent with the findings of (Marcos-Zambrano et al., 2014) who reports a strong production of biofilms in 80% of isolates of *C. tropicalis*; studies by (Guembe et al., 2017) report similar results indicating that biofilm production varies according to the *Candida* species and the physiology of the infection site; the species of *C. tropicalis* in our study were isolated from the urinary and respiratory tracts, which agrees with what was reported by (El-Kholy et al., 2021) where the isolates of *C. tropicalis* from blood, urinary and respiratory tracts produced a strong biofilm biomass. In our study *C. glabrata* had less biofilm production similar to that reported by (El-Kholy et al., 2021).

Our results report antibiofilm activity of ISO (IC_50_) against isolates of *C. tropicalis* 84, showing biofilm reduction percentages of 23.25% after 1 h of treatment, exhibiting a greater effect than AFB for some isolates. Likewise, the ISO (IC_50_) showed a 20% reduction of biofilms in *C. glabrata*, for which AFB had no effect (Figure 5(b)). shows the percentage of biofilm reduction after 1 h of treatment with ISO (MIC μg/mL) and AFB (1.0 μg/mL). The Mann-Whitney U test with a p-value > 0.05 and a confidence level of 95% tells us that there are no statistically significant differences between the effects of ISO and AFB on biofilm reduction. For *C. tropicalis* 81 and 107 an insignificant reduction of biofilms was observed (2.6 and 3% respectively) while for *C. auris* 75 and *C. albicans* (80, 83, 86) there was no reduction of biofilms, during the first hour of treatment. The ISO biofilm reduction percentages varied between species and between members of the same species, which is consistent with what was reported by (Prażyńska et al., 2018), which indicates that the inhibition of fungal biofilms may depend on the species and the degree of maturation of the biofilms. It should be noted that this is the first report of the effect of ISO on fungal biofilms in *Candida* spp. Regarding the treatment with AFB, detachment of biofilms was observed after 1 h of exposure in *C. auris* 75 (13.31%), *C. tropicalis* 84 (12%), *C. albicans* 86 (12.6%) and *C. tropicalis* 107 (5.11%), while for the rest of isolates it did not show reduction in the percentage of biofilms, during the first hour of treatment. Statistical analysis shows that there are no statistically significant differences between the effect of ISO and AFB against the reduction of fungal biofilms, however, our results show (Figure 5(b)) the effect of ISO on the biofilms of *C. tropicalis* (81 and 92), *C. albicans* (78 and 02) and *C. glabrata*, where AFB showed no effect.

### 3.6 Release of cellular material through the fungal membrane

**Figure 6:**
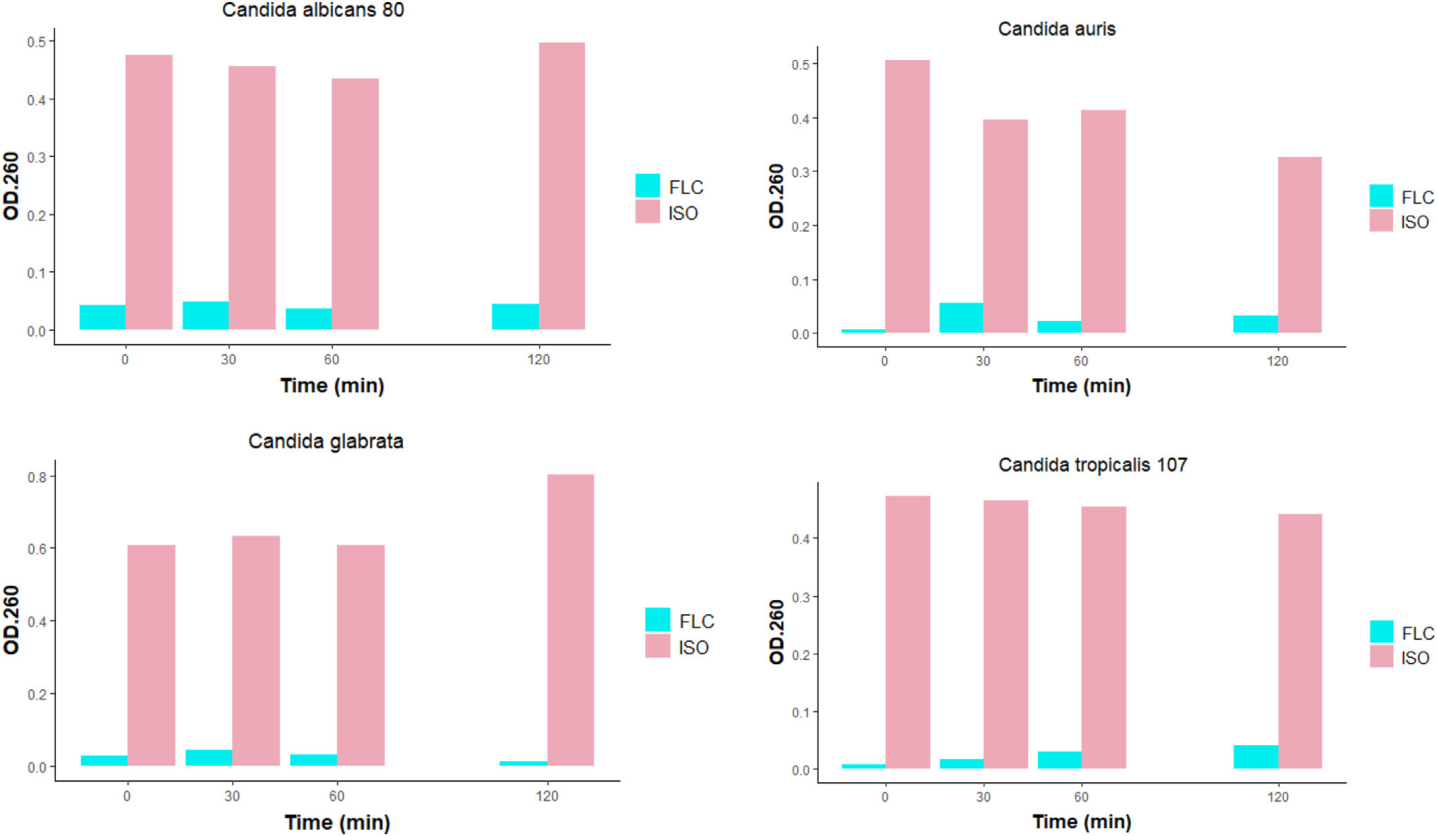
Effect of ISO and FLC on the release of intracellular components at 260 nm versus time. Significant differences are observed between the release of intracellular material from cells treated with ISO and cells treated with FLC.

The effect of ISO on the integrity of the *Candida* spp. membrane, and the release of cellular material at 0, 30, 60 and 120 min after ISO treatment was determined (Figure 6). The results are expressed as the absorbance of the sample (treated with ISO) minus the absorbance of the control (samples without ISO). The Student's T test with 95% confidence shows statistically significant differences between the effects of ISO and FLC against all *Candida* spp., in this study. Our results show a significant and marked, early release of cell content 1 h after ISO treatment compared to FLC-treated cells and untreated cells in all isolates evaluated in this study, probably as a result of damage to the cytoplasmic membrane, altering the permeability and causing leakage of intracellular material; the effect on the function and structure of the membrane has been generally used to explain the antimicrobial action of terpenes; as a result of their lipophilic character, they can interact with the membrane resulting in expansion, increased fluidity and permeability of the membrane, alteration of proteins embedded in the membrane, inhibition of respiration and alteration of the ion transport process as reported by (Trombetta et al., 2005).

### 3.7 Extracellular pH

**Figure 7:**
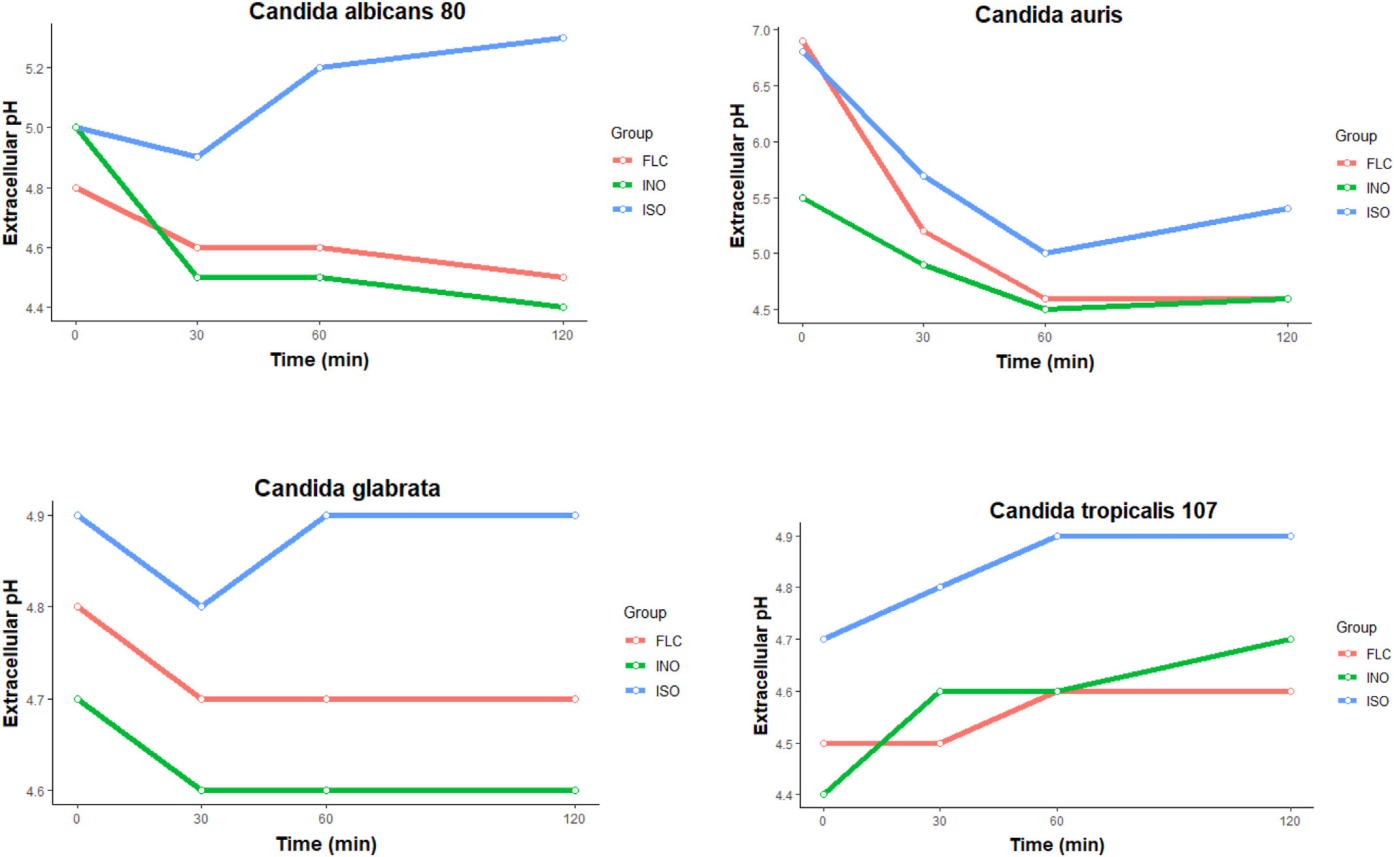
Extracellular pH of *Candida* spp., treated with ISO, FLC and untreated cells (INO). Important significant differences are observed between the increase in pH of the ISO-treated cells compared to the FLC-treated cells and the untreated cells.

The extracellular pH of the fungal cells treated with ISO, FLC and cells not treated is shown in Figure 7. The extracellular pH in *C. auris* decreased in the first 60 min and then increased unlike untreated cells and the cells with FLC; while the extracellular pH in *C. albicans* and *C. glabrata* decreased in the first 30 min, to then significantly increase in the cells treated with ISO unlike the cells treated with FLC and the cells without treatment. In contrast, the extracellular pH of *C. tropicalis* showed a considerable increase in the first 30 min and was constantly increasing. In the species of *C. albicans*, *C. glabrata* and *C. tropicalis*, the analysis of variance shows statistically significant differences between the averages of extracellular pH of the different groups, the honest Tukey test with 95% confidence shows significant differences between the extracellular pH means of the ISO-treated groups and the other groups (FLC-treated and untreated). With *C. auris*, although differences are observed, according to the analysis of variance with 95% confidence, these differences are not statistically significant, with a p-value > 0.05. In all cases, the extracellular pH of the cells treated with ISO is superior to the values obtained with the cells treated with fluconazole and those not treated. As we can observe, our results evidenced the early intracellular loss of protons, which is reflected with an increase in extracellular pH, more than that presented by the cells treated with fluconazole and the cells without treatment. Hyperpolarization has been reported as an important type of damage to the membrane and occurs mainly due to changes in pH or increase in ion movement affecting cellular homeostasis as reported by (Shi et al., 2016), it is our intention to suggest that the ISO powder alters the integrity of the membrane exchanging homeostasis, allowing the loss of ions and release of the cellular content, which could also be responsible for antifungal activity.

### 3.8 Integrity of the cellular membrane

The Evans blue staining also showed damage to the integrity of the membrane similar to the studies reported by (Donadu et al., 2021) with essential oil of *Ruta graveolens* against nosocomial cephalopods; studies of (Trombetta et al., 2005) suggest that this effect seems to depend on the composition of lipids and the net surface charge of the microbial membranes.

**Figure 8:**
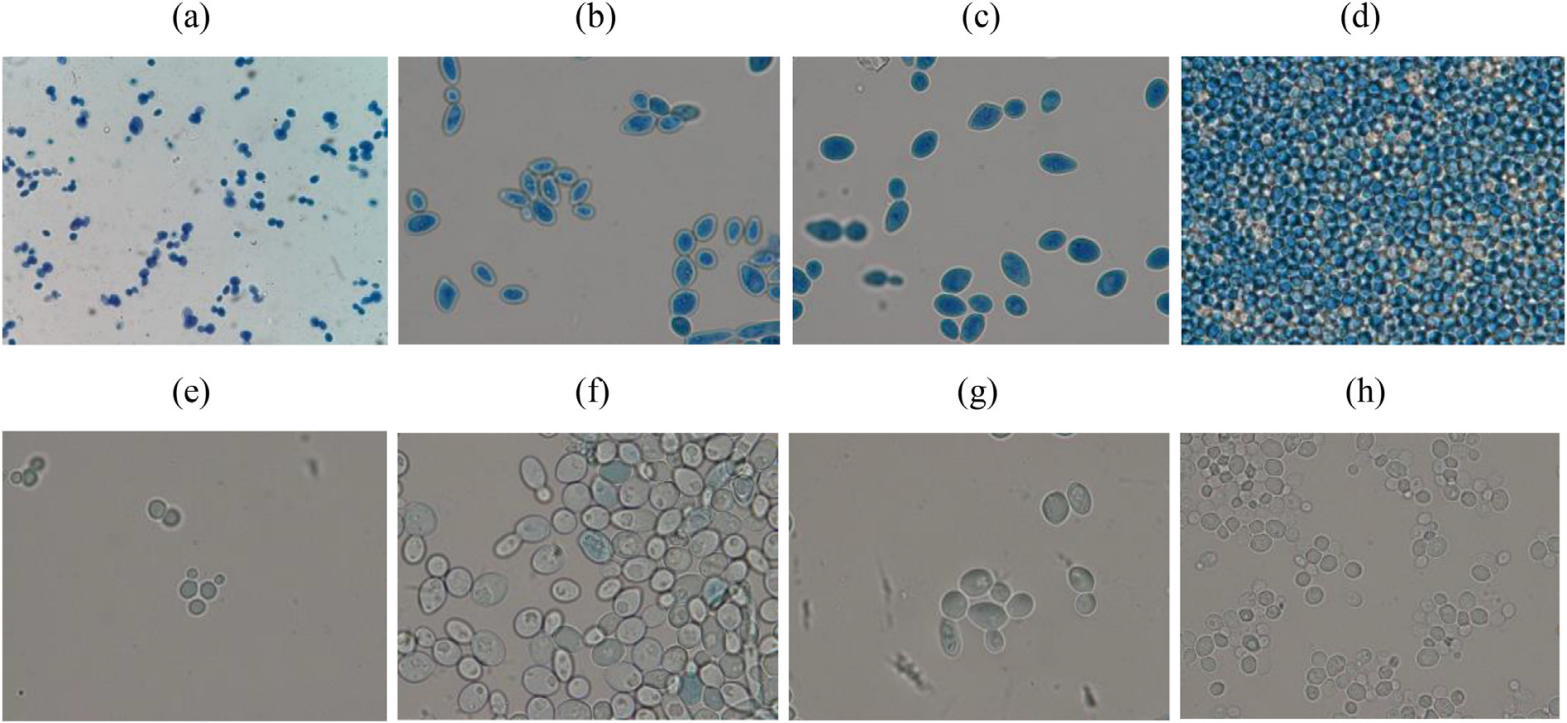
Microscopic observation (100x) of *C. albicans* (a), *C. tropicalis* (b), *C. glabrata* (c) and *C. auris* (d) before and after treatment with ISO: (a, b, c, d) cells treated with ISO (MIC) and control samples, not treated (e, f, g, h).

As indicated in Figure 8, the results show that when the cells were treated with ISO and observed under the light microscope, most of the cells stained blue, suggesting that the cell membranes were compromised after 1 h of treatment with ISO, therefore the ISO could act on the membrane affecting its integrity and consequently resulting in an increase in intracellular leakage of macromolecules, so we could suggest that the plasma membrane is a target of the ISO mode of action against *Candida* spp.

Our study revealed the antifungal effect of ISO against clinical isolates of Candida and highlights its possible potential usefulness as a component of new antifungals or as an adjunct in the treatment of infections caused by these yeasts. In addition, our results serve as the basis for future studies with a view to expanding our knowledge, establishing the mechanisms of antifungal action of the ISO.

## 4 Conclusions

In conclusion, the results found allow us to propose that the antifungal activity of ISO is due, at least partially, to the fact that this molecule is capable of disturbing the integrity of fungal plasma membranes, resulting in the alteration of the permeability of the membrane and the consequent loss of intracellular material. Therefore, ISO is a natural chemical compound with antifungal properties.

## Declarations

### Author contribution statement

Orfa Inés Contreras Martínez; Alberto Angulo Ortíz; Gilmar Santafé Patiño: Conceived and designed the experiments; Performed the experiments; Analyzed and interpreted the data; Contributed reagents, materials, analysis tools or data; Wrote the paper.

### Funding statement

Orfa Inés Contreras Martínez was supported by Universidad de Córdoba (FCB-02-19 project).

### Data availability statement

Data will be made available on request.

### Declaration of interest’s statement

The authors declare no conflict of interest.

### Additional information

No additional information is available for this paper.

## References

[bib1] Arango N., Vanegas N., Sáez J., García C., Rojano B. (2007). Actividad antifúngica del isoespintanol sobre hongos del género Colletotricum. Sci. Tech..

[bib2] Avato P. (2020). Editorial to the special Issue –“ natural products and drug discovery. Molecules.

[bib3] Aylate A., Agize M., Ekero D., Kiros A., Ayledo G., Gendiche K. (2017). In-Vitro and In-Vivo Antibacterial Activities of Croton Macrostachyus Methanol Extract against E. coli and S. aureus. Adv. Anim. Vet. Sci..

[bib4] Bongomin F., Gago S., Oladele R., Denning D. (2017). Global and multi-national prevalence of fungal diseases-estimate precision. J. Fungi.

[bib5] Byvaltsev V.A., Bardonova L.A., Onaka N.R., Polkin R.A. (2019). Acridine orange : a Review of Novel Applications for surgical cancer imaging and therapy. Front. Oncol..

[bib6] Cantón E., Martín E., Espinel-Ingroff A. (2007).

[bib7] Carvajal S.K., Alvarado M., Rodriguez Y.M., Parra-Giraldo C.M., Varón C., Morales S.E., Rodriguez J., Gómez B.L., Escandón P. (2021). Pathogenicity assessment of Colombian strains of *Candida auris* in the *Galleria mellonella* invertebrate model. J. Fungi.

[bib8] Chaves-Lopez C., Nguyen H.N., Oliveira R.C., Nadres E.T., Paparella A., Rodrigues D.F. (2018). A morphological, enzymatic and metabolic approach to elucidate apoptotic-like cell death in fungi exposed to h- and α-molybdenum trioxide nanoparticles. Nanoscale.

[bib9] Chen P., Chuang Y., Wu U., Sun H., Wang J., Sheng W., Chen Y., Chang S. (2021). Mechanisms of azole resistance and trailing in *Candida tropicalis* Bloodstream isolates. J. Fungi.

[bib10] Cheng R., Xu Q., Hu F., Li H., Yang B., Duan Z., Zhang K., Wu J., Li W., Luo Z. (2021). Antifungal activity of MAF-1A peptide against *Candida albicans*. Int. Microbiol..

[bib11] De Oliveira Lima M.I., Araújo de Medeiros A.C., Souza Silva K.V., Cardoso G.N., de Oliveira Lima E., de Oliveira Pereira F. (2017). Investigation of the antifungal potential of linalool against clinical isolates of fluconazole resistant *Trichophyton rubrum*. J. Mycol. Med..

[bib12] De Oliveira Pereira F., Moura Mendes J., De Oliveira Lima E. (2013). Investigation on mechanism of antifungal activity of eugenol against *Trichophyton rubrum*. Med. Mycol..

[bib13] Dias de Castro R., Souza P. A. de, Dornelas Bezerra L., Silva Ferreira G., Melo de Brito Costa E., Leite Cavalcanti A. (2015). Antifungal activity and mode of action of thymol and its synergism with nystatin against Candida species involved with infections in the oral cavity: an *in vitro* study. BMC Compl. Alternative Med..

[bib14] Donadu M.G., Peralta-Ruiz Y., Usai D., Maggio F., Molina-Hernandez J.B., Rizzo D., Bussu F., Rubino S., Zanetti S., Paparella A., Chaves-Lopez C. (2021). Colombian essential oil of *Ruta graveolens* against nosocomial antifungal resistant Candida strains. Journal of Fungi.

[bib15] El-Kholy M.A., Helaly G.F., El Ghazzawi E.F., El-Sawaf G., Shawky S.M. (2021). Virulence factors and antifungal susceptibility profile of *C . Tropicalis* isolated from various clinical specimens in. J. Fungi.

[bib16] Gavilánez T.C., Colareda G.A., Ragone M.I., Bonilla M., Rojano B.A., Schinella G.R., Consolini A.E. (2018). Intestinal, urinary and uterine antispasmodic effects of isoespintanol, metabolite from *Oxandra xylopioides* leaves. Phytomedicine.

[bib17] Gintjee T.J., Donnelley M.A., Thompson G.R. (2020). Aspiring antifungals : Review of current antifungal pipeline developments. J. Fungi.

[bib18] Guembe M., Cruces R., Peláez T., Mu P., Bouza E. (2017). Assessment of biofilm production in Candida isolates according to species and origin of infection. Enferm. Infecc. Microbiol. Clín..

[bib19] Hassan Y., Chew S.Y. (2021). Candida glabrata : pathogenicity and resistance mechanisms for adaptation and survival. Journal of Fungi.

[bib20] Janbon G., Quintin J., Lanternier F., Enfert D. (2019). Studying Fungal Pathogens of Humans and Fungal Infections : Fungal Diversity and Diversity of Approaches. Gene Immun..

[bib21] Kakar A., Holzknecht J., Dubrac S., Gelmi M.L., Romanelli A. (2021). New perspectives in the antimicrobial activity of the Amphibian Temporin B : peptide Analogs are effective Inhibitors of *Candida albicans* growth. J. Fungi.

[bib22] Marcos-zambrano L.J., Escribano P., Bouza E., Guninea J. (2014). Production of biofilm by Candida and non- Candida spp. isolates causing fungemia : Comparison of biomass production and metabolic activity and development of cut-off points. Int. J. Med. Microbiol..

[bib23] Mekonnen Bayisa Y., Aga Bullo T. (2021). Optimization and characterization of oil extracted from *Croton macrostachyus* seed for antimicrobial activity using experimental analysis of variance. Heliyon.

[bib24] Mukherjee P.K., Long L., Kim H.G., Ghannoum M.A. (2009). International Journal of Antimicrobial Agents Amphotericin B lipid complex is efficacious in the treatment of *Candida albicans* biofilms using a model of catheter-associated Candida biofilms. Int. J. Antimicrob. Agents.

[bib25] Murphy Cowan M. (1999). Plant products as antimicrobial Agents. Clin. Microbiol. Rev..

[bib26] Naman C.B., Benatrehina P.A., Kinghorn A.D. (2016). Encyclopedia of Applied Plant Sciences.

[bib27] Oliveira Lima I., de Oliveira Pereira F., Araújo de Oliveira W., de Oliveira Lima E., Albuquerque Menezes E. (2013). Antifungal activity and mode of action of carvacrol against *Candida albicans* strains. J. Essent. Oil Res..

[bib28] Prażyńska M., Bogiel T., Gospodarek-Komkowska E. (2018). *In vitro* activity of micafungin against biofilms of *Candida albicans* , *Candida glabrata* , and *Candida parapsilosis* at different stages of maturation. Folia Microbiol..

[bib29] Quave C.L., Plano L.R.W., Pantuso T., Bennett B.C. (2008). Effects of extracts from Italian medicinal plants on planktonic growth, biofilm formation and adherence of methicillin-resistant *Staphylococcus aureus*. J. Ethnopharmacol..

[bib30] Ramírez R.D., Páez M.S., Angulo A.A. (2015). Obtención de isoespintanol por hidrodestilación y cristalización a partir del extracto bencínico de *Oxandra xylopioides*. Inf. Tecnol..

[bib31] Rinaldi G.J., Rojano B., Schinella G., Mosca S.M. (2019). Participation of NO in the vasodilatory action of isoespintanol. Vitae.

[bib32] Rodriguez-Tudela J.L. (2003). Method for the determination of minimum inhibitory concentration (MIC) by broth dilution of. Clin. Microbiol. Infect., August.

[bib33] Rojano B.A., Gaviria C.A., Gil M.A., Sáez J.A., Schinella G.R., Tournier H. (2008). Antioxidant activity of the isoespintanol in diferent media. Vitae.

[bib34] Rojano B.A., Montoya S., Yépez F., Saez J. (2007). Evaluación de isoespintanol aislado de *Oxandra cf. xylopioides* (Annonaceae) sobre *Spodoptera frugiperda* J.E. SMITH (Lepidoptera: noctuidae). Rev. Fac. Nac. Agron. Medellin.

[bib35] Rojano B., Pérez E., Figadère B., Martin M.T., Recio M.C., Giner R., Ríos J.L., Schinella G., Sáez J. (2007). Constituents of *Oxandra cf. xylopioides* with anti-inflammatory activity. J. Nat. Prod..

[bib36] Scorneaux B., Angulo D., Borroto-Esoda K., Ghannoum M., Peel M., Wring S. (2017). SCY-078 is fungicidal against Candida species in time-kill studies. Antimicrob. Agents Chemother..

[bib37] Shi C., Sun Y., Zheng Z., Zhang X., Song K., Jia Z., Chen Y., Yang M., Liu X., Dong R., Xia X. (2016). Antimicrobial activity of syringic acid against *Cronobacter sakazakii* and its effect on cell membrane. Food Chem..

[bib38] Tao N., Ouyang Q., Jia L. (2014). Citral inhibits mycelial growth of *Penicillium italicum* by a membrane damage mechanism. Food Control.

[bib39] Tascini C., Sozio E., Corte L., Sbrana F. (2017). The role of biofilm forming on mortality in patients with candidemia : a study derived from real world data. Infect. Dis..

[bib40] Trombetta D., Castelli F., Sarpietro M.G., Venuti V., Cristani M., Daniele C., Saija A., Mazzanti G., Bisignano G. (2005). Mechanisms of antibacterial action of three monoterpenes. Antimicrob. Agents Chemother..

[bib41] Wu G.P., Chen S.H., Levin R.E. (2015). Application of ethidium bromide Monoazide for quantification of viable and dead cells of *Salmonella enterica* by real-time loop-mediated isothermal amplification. J. Microbiol. Methods.

[bib42] Zhang X., Zhang T., Guo S., Zhang Y., Sheng R., Sun R., Chen L., Lv R., Qi Y. (2020). *In vitro* antifungal activity and mechanism of Ag3PW12O40 composites against Candida species. Molecules.

